# Impact of multiple-scale circulation interactions on the spring diurnal precipitation over Luzon

**DOI:** 10.1038/s41598-021-89392-0

**Published:** 2021-05-11

**Authors:** Cheng-An Lee, Wan-Ru Huang, Ya-Hui Chang, Shih-Ming Huang

**Affiliations:** 1 Department of Earth Sciences, National Taiwan Normal University, Taipei, Taiwan; 2 Department of Atmospheric Sciences, Chinese Culture University, Taipei, Taiwan

**Keywords:** Climate sciences, Environmental sciences, Hydrology

## Abstract

This study examines the spatiotemporal characteristics of diurnal precipitation over Luzon and the nearby oceans in boreal spring. The study focuses on exploring the impact of the interaction between large- and local-scale circulation changes on the modulation of diurnal precipitation. We analyze the satellite precipitation data obtained from the Tropical Rainfall Measuring Mission and Global Precipitation Measurement Mission during the spring (March–May) of 2001–2019. The results show that the spring diurnal precipitation over Luzon and the nearby oceans consists of a clear eastward propagation signal. The direction of this propagation is opposite to that of the prevailing low-level easterly wind in spring and differs from the well-known westward propagation direction of diurnal precipitation over Luzon in summer. Diagnoses of the possible maintenance mechanisms suggest that the eastward propagation diurnal precipitation can be attributed to the interaction between the topography and multiple-scale circulation changes, including the mountain–valley breeze, island-scale land–sea breeze (LSB), large-scale LSB-like circulation, and mid-to-upper-level prevailing wind fields. This finding highlights the importance of considering multiple-scale circulation changes in the modulation of spring diurnal precipitation over the East Asia–western North Pacific region.

## Introduction

**Figure 1:**
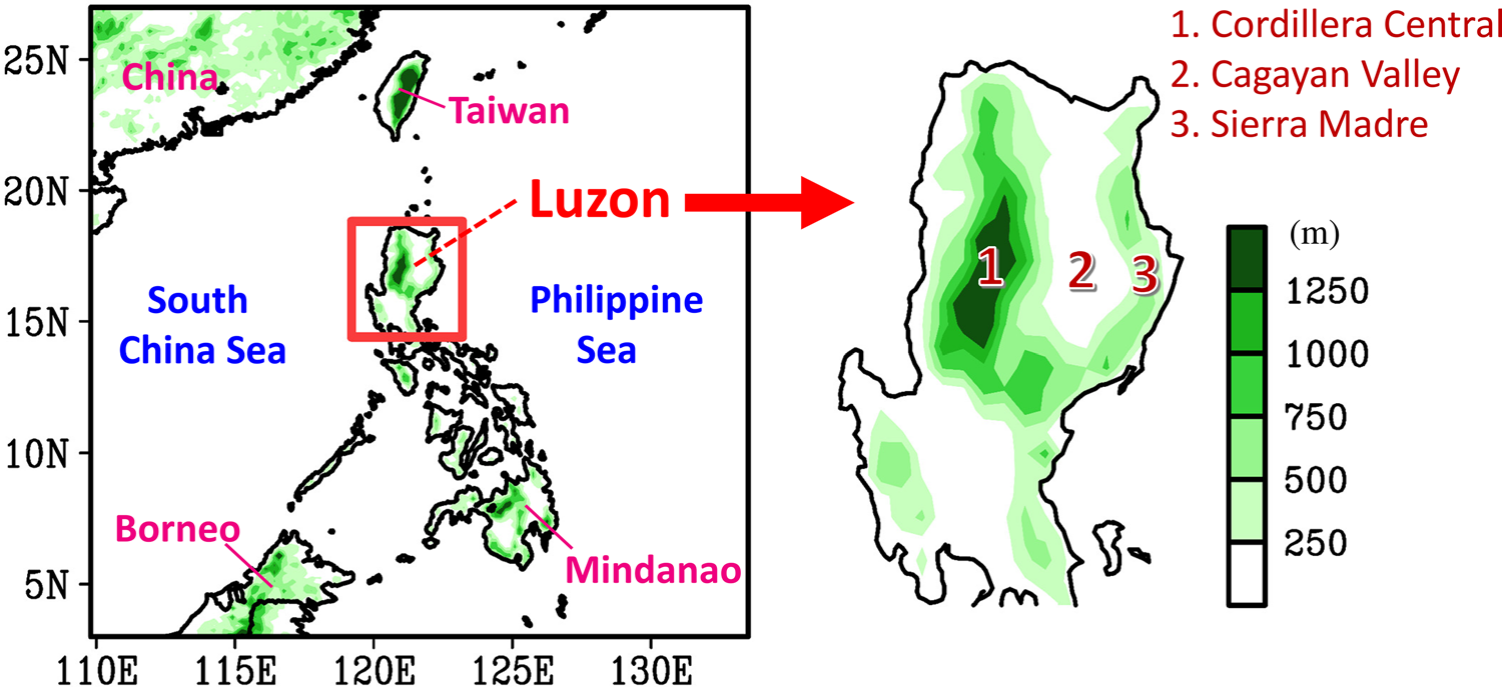
Geographic locations and topography over East Asia (left panel) and Luzon (right panel). This figure is created using the software of Grid Analysis and Display System version 2.1.1.b0 (hereafter GrADS v2.1.1.b0), available at http://cola.gmu.edu/grads/downloads.php.

Luzon is the largest island in the Philippines, located in the East Asia–western North Pacific (EAWNP) region. The topography of Luzon is characterized by the Cordillera Central (north–south-oriented mountain) in the west, the Sierra Madre (north–south-oriented mountain) in the east, and the Cagayan Valley between the two mountain ranges (Fig. 1). Climatologically, the spatial distribution of seasonal mean precipitation over Luzon is affected by the interaction between the topography and seasonal wind fields, which results in a larger seasonal mean precipitation in the windward regions^1–5^. For example, in summer (June, July, and August; JJA), Luzon is affected by low-level southwesterly monsoon, with a larger precipitation concentrated in the west^4,5^. In contrast, in winter (December, January, and February; DJF), Luzon is affected by low-level northeasterly monsoonal winds, with a larger precipitation concentrated in the east^3,6^. In spring (March, April, and May; MAM) and autumn (September, October, and November; SON), the low-level easterly wind prevails over Luzon, with a larger precipitation concentrated in the east^1,2^.

In addition to seasonal variation, the precipitation over Luzon exhibits a diurnal variation^7–11^. Ho et al.^7^ examined the characteristics of summer diurnal precipitation over Luzon and found that the satellite precipitation observed from the Tropical Rainfall Measuring Mission (TRMM) had a maximum peak on land in the afternoon. The local convection subsequently propagated westward, leading to a maximum precipitation peak at midnight over the west coast and the nearby oceans. The westward propagation of summer diurnal precipitation over Luzon is possibly caused by the mutual adjustment of several related mechanisms summarized below. In mountainous areas over Luzon, earlier studies have indicated that diurnal precipitation is mainly caused by the convergence of sea breezes on the islands, and the valley breezes induced by solar thermal heating further enhance local convection^12–14^. In contrast, other studies have indicated that the combination of mountain and land breezes at nighttime can intensify outward divergence over Luzon. This can further cause the propagation of local convection from the inner land to coastal regions^11,15–19^. Moreover, it has been suggested that the upper-level prevailing easterly winds help propagate summer diurnal precipitation from the coastal regions to the ocean west of Luzon^7^.

The characteristics and maintenance mechanisms of diurnal precipitation over the EAWNP region in different seasons are possibly different^20–23^. For example, Huang and Chan^22^ examined the seasonal variation of diurnal precipitation over Southeast China; they found that the diurnal precipitation in winter peaks in the early morning (05 h; local time), which is different from the diurnal precipitation maximum occurring in the late afternoon (17 h) in other seasons. Other studies examining diurnal precipitation in Taiwan have also noted seasonal differences^23,24^. For example, Huang and Wang^24^ examined diurnal precipitation in Taiwan during May and June and found that the precipitation had a peak value in the late afternoon (17 h) and moved eastward. In contrast, Huang and Chang^23^ found that the winter diurnal precipitation in Taiwan had two peak values, one at night (22 h) and another in the early morning (06 h). As Luzon is located near Southeast China and Taiwan, it is likely that diurnal precipitation over Luzon displays seasonal differences. Previous studies on diurnal precipitation over Luzon mostly focused on the summer season^7–11^ and rarely investigated other seasons.

**Figure 2:**
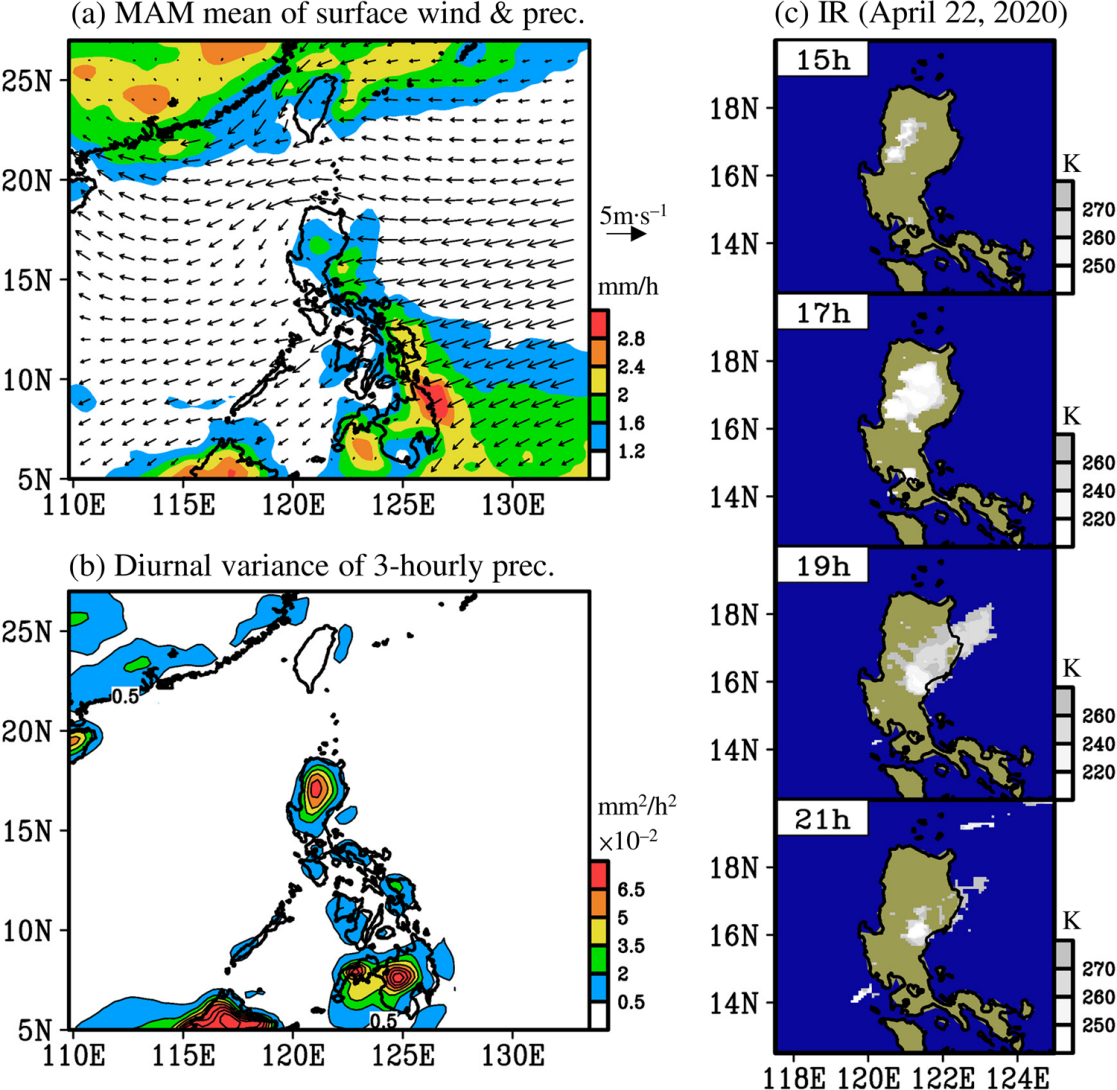
(**a**) Seasonal mean of surface wind (vectors) extracted from ERA5, superimposed with precipitation extracted from TRMM, and averaged for spring during 2001–2019 (March to May; MAM). (**b**) Diurnal variance of three-hourly precipitation estimated by TRMM, averaged during 2001–2019 spring. (**c**) Infrared satellite imagery for an eastward propagation of spring diurnal precipitation that occurred on April 22, 2020; the IR image is created from Gridded Satellite B1 observations ( available at https://www.ncdc.noaa.gov/gridsat/). In (**c**), the times are given in local time. This figure is created using the software of GrADS v2.1.1.b0.

From a series of satellite images captured on April 22, 2020, we noted that the spring diurnal precipitation over Luzon and the nearby ocean appeared to propagate eastward, which is different from the well-known westward propagation of diurnal precipitation in summer and opposite to the prevailing seasonal low-level wind in spring (Fig. 2; discussed later). Therefore, motivated by this observation, two scientific objectives are examined in this study: (1) to determine whether the climatological diurnal precipitation over Luzon in spring shows eastward propagation, and (2) if eastward propagation is confirmed, to determine the mechanisms of formation.

For objective (2), possible mechanisms (including atmospheric circulation modulations^7–11,19,20,23,24^, gravity waves^16,25^, and cold pool outflow^11,26^) may be inferred from earlier studies that examined diurnal precipitation over the EAWNP region. In this study, we are mainly interested in the mechanisms related to the referenced atmospheric circulation modulations. Huang and Wang^24^ suggested that the eastward propagation of diurnal precipitation over Taiwan was induced by the interaction between island-scale land–sea breeze (LSB) and large-scale LSB-like circulation over the EAWNP region. The diurnal precipitation over Luzon in spring might be affected by a similar physical mechanism. This hypothesis is examined in this study. On the other hand, Ho et al.^7^ suggested that the westward propagation of summer diurnal precipitation over Luzon was affected by the prevailing easterly wind at the upper level. The diurnal precipitation over Luzon in spring might be affected by the prevailing wind at certain levels. This hypothesis is also examined in this study.

## Data and methods

The precipitation data used in this study were obtained from two sources, TRMM 3B42 v7 (TRMM)^27^ and Integrated Multi-satellitE Retrievals for Global Precipitation Measurement Final run v6 (hereafter IMERG)^28,29^. TRMM (three-hourly, 0.25° × 0.25° resolution) is one of the most frequently used datasets for studying precipitation changes over the EAWNP region^30^. Recently, Sunilkumar et al.^31^ analyzed the characteristics of precipitation over the Philippines and proved that IMERG (30 min, 0.1° × 0.1° resolution), which was released by the National Aeronautics and Space Administration in March 2014 for global precipitation monitoring, represents surface precipitation measurements better than TRMM. Therefore, in addition to the frequently used TRMM data, we used the latest IMERG data to investigate the characteristics of precipitation over Luzon and the nearby oceans. This analysis focused on boreal spring months (MAM) of 2001–2019, for which both TRMM and IMERG datasets were available.

To explore the possible mechanisms of precipitation changes, we used meteorological variables (including wind fields, vertical velocity, surface temperature, and sea level pressure) provided by the European Centre for Medium-Range Weather Forecasts global climate reanalysis fifth generation (ERA5)^32,33^. The ERA5 has a spatial resolution of 0.25° × 0.25° and a temporal resolution of 1 h, which is finer than most other global reanalyses and suitable for investigating diurnal variation. Hereafter, all analyses refer to the local time in the Philippines, which is universal time (UTC) + 8 h. The climatological features were obtained by averaging the time periods of 2001–2019 for MAM. The diurnal anomalies of a selected variable during a day were obtained by removing the daily mean from the variable.

## Results

### Spatiotemporal characteristics of diurnal precipitation in spring

Figure 2a shows the spatial distribution of seasonal mean precipitation in spring over the EAWNP region. Due to the interaction between the topography and seasonal low-level easterly winds over Luzon, a larger amount of precipitation is concentrated in the eastern Luzon and the nearby ocean. In addition to this seasonal feature, precipitation over Luzon consists of clear diurnal variability in spring, as shown in Fig. 2b. On April 22, 2020, a series of infrared satellite images showed that local clouds had started to form in the Cordillera Central at 15 h, which then developed and moved eastward to the Cagayan Valley and the Sierra Madre, and finally disappeared over eastern Luzon and the nearby ocean at 21 h (Fig. 2c). This eastward propagation of diurnal precipitation is opposite of the prevailing low-level easterly wind (Fig. 2a) and different from the well-known westward propagation of diurnal precipitation in summer^8,10,11^. Therefore, the question arises whether the observed eastward propagation of diurnal precipitation in Fig. 2c is a special case or a common feature during spring.

**Figure 3:**
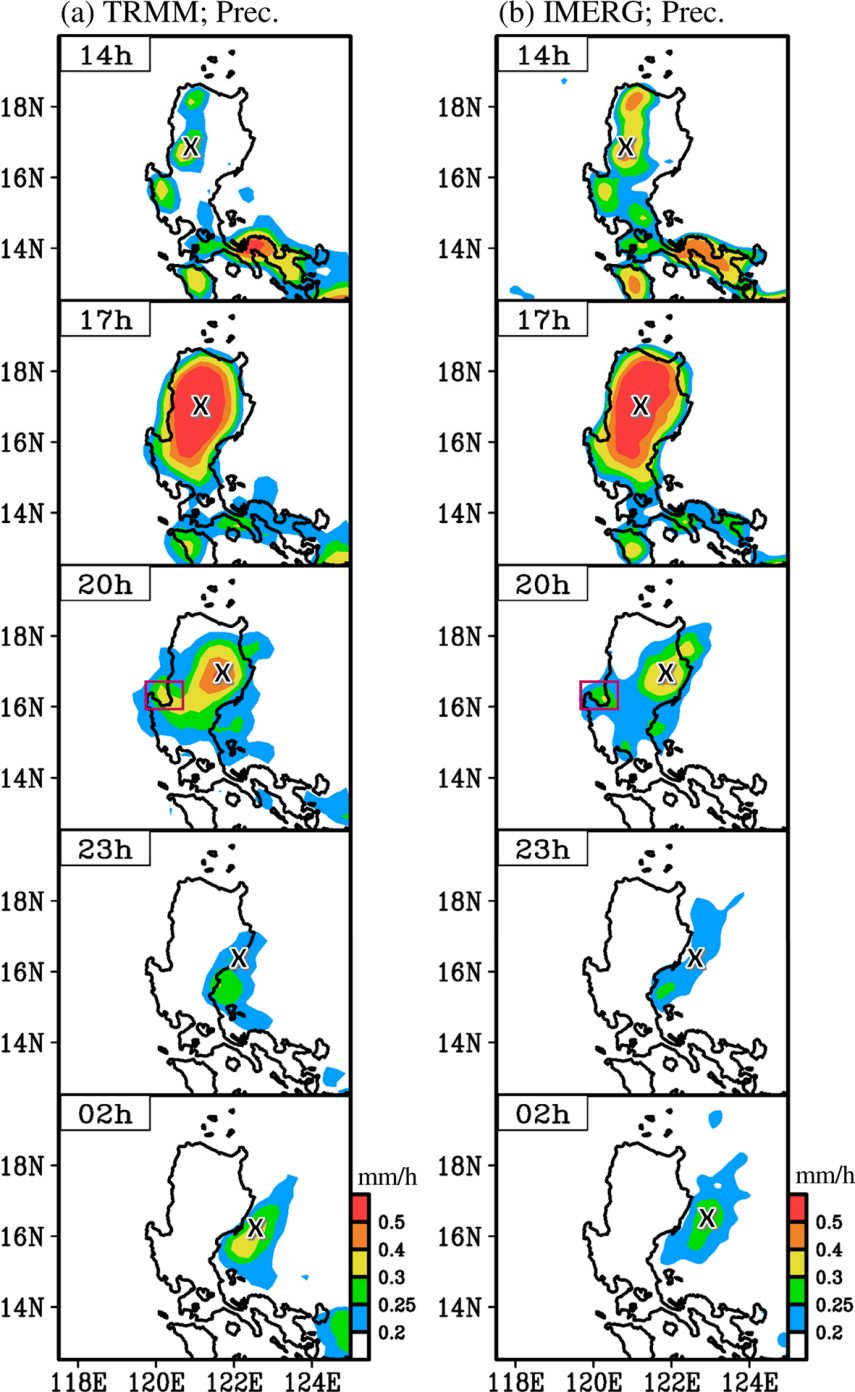
Horizontal distribution of three-hourly precipitation at selected timesteps during a day in spring, averaged during 2001–2019, estimated by: (**a**) TRMM and (**b**) IMERG. The times (14–02 h) represent the local time in the Philippines. The symbol “x” indicates the eastward propagation of diurnal precipitation, while the open square at 20 h is added to clarify the discussion. This figure is created using the software of GrADS v2.1.1.b0.

To clarify the above question, we examined the spatiotemporal variation of the climatology (2001–2019 mean) of diurnal precipitation over Luzon in spring. The climatological diurnal precipitation was obtained from TRMM (Fig. 3a) and IMERG (Fig. 3b) during 2001–2019 for MAM. As shown in Fig. 3a, the climatological diurnal precipitation began to appear in western Luzon over the Cordillera Central at 14 h, which then enhanced and expanded eastward to cover most of Luzon, with the maximum diurnal precipitation occurring at 17 h. Subsequently, the location of precipitation moved eastward over the Cagayan Valley and the Sierra Madre at 20 h, while the precipitation over the Cordillera Central weakened at 20 h compared to that at 17 h. Although the precipitation over western Luzon and the nearby ocean disappeared at 23 h–02 h, the precipitation over eastern Luzon and the nearby ocean persisted and even slightly enhanced and expanded eastward. Notably, despite the difference in magnitude, the characteristics of precipitation propagation revealed in IMERG (Fig. 3b) are similar to those in TRMM (Fig. 3a), showing that the climatological spring diurnal precipitation over Luzon and the nearby oceans is mainly characterized by an eastward propagation feature.

**Figure 4:**
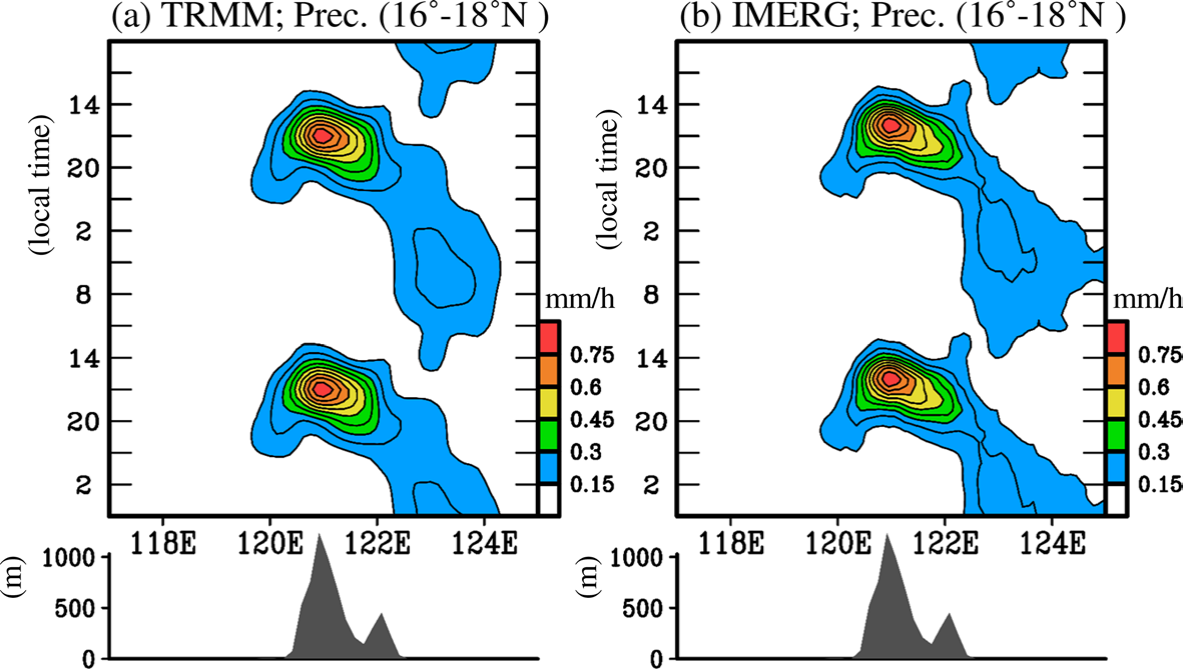
Longitude (x-axis) versus time (y-axis) diagram of climatological diurnal evolution of precipitation averaged over 16–18°N for spring during 2001–2019: (**a**) TRMM and (**b**) IMERG. The times are given in local time. The altitude of topography averaged over 16–18°N is given in the bottom panel to help the discussion. This figure is created using the software of GrADS v2.1.1.b0.

Figure 3 only shows features for certain selected time steps during a day; therefore, we constructed a longitude-time diagram of precipitation averaged over 16–18°N (Fig. 4) to better illustrate the features of propagation for all time steps during a day. The altitude of the topography averaged over 16–18°N is also demonstrated in Fig. 4 to facilitate discussion. As noted from TRMM (Fig. 4a), climatological precipitation during a day began to appear in western Luzon around 14 h and reached a maximum value over the Cordillera Central at 17 h. During 17–23 h, most of the precipitation moved eastward to the Cagayan Valley and the Sierra Madre, while a small part of the precipitation spread westward to 120°E near the western coast. Corresponding to this feature, in Fig. 3, a small precipitation center can be seen around 16°N, 120°E at 20 h (marked by an open square), which is located west of the maximum precipitation center at 17 h. The above-mentioned maximum precipitation over most of Luzon and the ocean near western Luzon disappeared during 02–08 h. In contrast, precipitation over the ocean near eastern Luzon not only persisted but also enhanced around 02–08 h.

In addition to TRMM (Fig. 4a), IMERG (Fig. 4b) also depicts similar propagation features, especially over the land areas of Luzon. The major difference between TRMM (Fig. 4a) and IMERG (Fig. 4b) can be seen over the ocean near eastern Luzon between 122 and 124°E, where the enhancement of diurnal precipitation occurred during 23–08 h in IMERG, approximately 3 h earlier than that in TRMM. Despite the difference, it is obvious that both TRMM and IMERG show climatological diurnal precipitation over Luzon, which is mainly characterized by the eastward propagation feature, suggesting that Fig. 2c represents a typical case, not a special case.

The cause of the eastward propagation of spring diurnal precipitation over Luzon and the nearby oceans must be known. Earlier studies have suggested that the variation in diurnal precipitation over East Asia is modulated by several factors, including atmospheric circulation changes, thermal instability changes, and gravity wave changes^15,18,20,24–26,34,35^. In this study, we are mainly interested in understanding whether the propagation observed by TRMM and IMERG (Figs. 3–4) can be explained by the interaction between the topography and atmospheric circulation changes. In view of earlier literatures, the atmospheric circulation changes that possibly play a role in modulating the diurnal precipitation formation include: (i) the island-scale LSB^12,13,15^, (ii) mountain–valley breeze^9,14,16,19^, (iii) large-scale LSB over the EAWNP region^24^, and (iv) prevailing wind field at upper level^7,11,16^. In the next section, we verify how these factors affect diurnal precipitation formation over Luzon. However, to avoid redundancy, only the precipitation from IMERG (with higher spatiotemporal resolution) was employed for the analyses.

### Atmospheric circulation modulations for diurnal precipitation propagation

First, we constructed the climatological (2001–2019 MAM mean) horizontal distribution of the near-surface wind field and related wind convergence for certain selected time steps during a day (Supplementary Fig. S1a). When focusing on the positive values of wind convergence in Fig. S1a, a clear eastward propagation feature is observed over Luzon (16–18°N). Positive values of wind convergence can help provide more dynamical lifting to facilitate precipitation formation^22–24^. As inferred from this general concept, the low-level atmospheric circulation modulations during a day (Fig. S1a) likely play an important role in causing the eastward propagation of diurnal precipitation over Luzon (Fig. 3). However, as shown in Fig. S1a, the magnitudes of wind vectors over Luzon were much smaller than those over the nearby oceans. Thus, it is not easy to express the local land circulation features (including island-scale LSB and mountain–valley breezes) within Fig. S1a.

To more thoroughly clarify the climatological low-level circulation changes during a day, we separated the original wind fields (Fig. S1a) into two components: daily mean (Fig. S1b) and diurnal anomalies (i.e., fluctuations; Fig. S1c)^24^. Overall, the magnitude of wind vectors was larger within the daily mean than the fluctuations over the ocean (Figs. S1b-c). However, when focused on only the land areas (Fig. S2), it is clear that the magnitudes of low-level wind vectors at 14 h were smaller within the daily mean than the fluctuations over the western Luzon. Specifically, at 14–17 h, the magnitude of the wind convergence calculated by the fluctuation component (Fig. S1c) is much larger than that calculated by the daily mean component (Fig. S1b). Spatially, the daily mean component mainly demonstrates that wind convergence existed over eastern Luzon (the windward side); this pattern is stationary from 14–02 h. In contrast, the fluctuation component induces a propagating wind convergence feature from 14–02 h. These features suggest that the eastward propagation of low-level wind convergence over Luzon (Fig. S1a) is mainly dominated by its fluctuation component (Fig. S1c) and less by its daily mean component (Fig. S1b). Based on these findings, the following discussions of maintenance mechanisms are mainly presented in the fluctuation component, which shows the details of the land circulation changes and the propagating feature (e.g., Fig. S1c).

**Figure 5:**
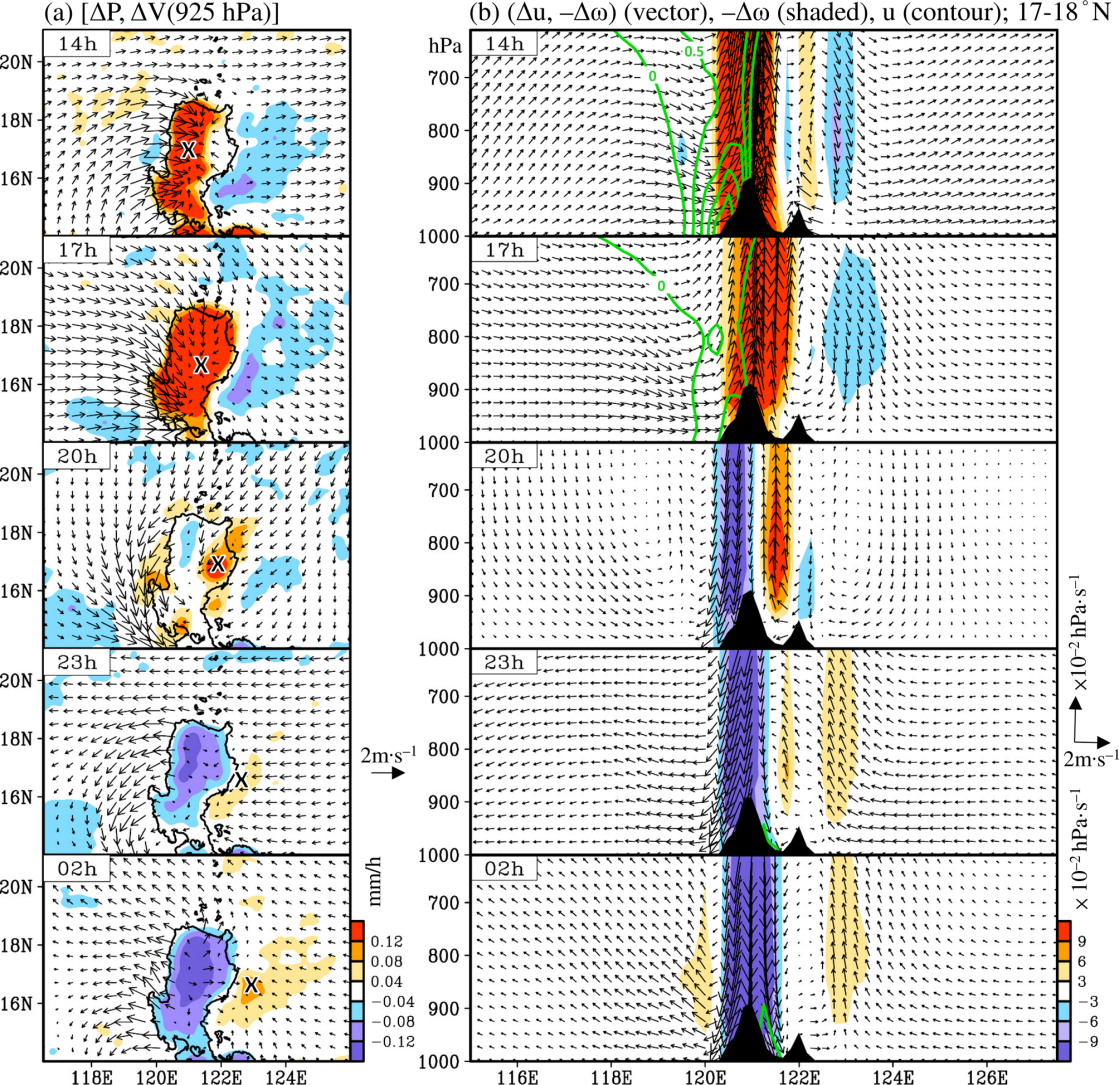
(**a**) Horizontal distribution of diurnal anomalies (daily mean removed, denoted as Δ) of precipitation (ΔP, shaded) extracted from IMERG superimposed with the low-level wind field at 925 hPa [ΔV (925 hPa), vector] extracted from ERA5 and averaged for spring from 2001–2019. The times (14–02 h) represent the local time in the Philippines. (**b**) is related to (a), but instead represents the vertical cross-section of the diurnal anomalies of longitudinal wind (Δu, horizontal vector) and vertical wind (− Δω, vertical vector) averaged over 17–18°N. In (**b**), the diurnal anomalies of the vertical velocity (− Δω; shaded) and the original longitudinal wind (u, only positive values of u are contoured; interval is 0.5 m/s) averaged over 17–18°N are added for additional clarity. The symbol “x” indicates the eastward propagation of diurnal precipitation. This figure is created using the software of GrADS v2.1.1.b0.

Causes for diurnal precipitation over western Luzon at 14 h were investigated. Figure 5a shows the climatological horizontal distribution of diurnal anomalies (i.e., daily mean removed) of the low-level wind field, which is superimposed with the diurnal anomalies of precipitation over Luzon. In addition, a related vertical cross-section of diurnal anomalies of east–west wind circulation (vector) averaged over 17–18°N (Fig. 5b) has been constructed to enhance this discussion. As shown in Fig. 5a, the appearance of precipitation over western Luzon at 14 h matches closely with the convergence center of the wind field. This convergence of low-level wind can further induce a stronger upward motion over the Cordillera Central in western Luzon (Fig. 5b), leading to precipitation formation over the region (Fig. 5a). Undoubtedly, low-level wind convergence plays an important role in modulating diurnal precipitation formation over Luzon. The reason for the east–west asymmetry in the low-level wind convergence over Luzon at 14 h must be determined. In general, the local circulation over Luzon, induced by the land–sea thermal contrast, is characterized by island-scale sea breezes during daytime^8,12,13^. However, the formation of island-scale sea breezes alone, without considering the effect of topography, should be symmetrical. Therefore, other factors also play a role in modulating the low-level wind convergence at 14 h over Luzon.

As noted from Fig. 5b, the formation of local valley breezes might be one of the factors. A taller mountain can lead to a larger mountain–valley breeze relative to a shorter mountain^9,14^. Because the altitude of the Cordillera Central is taller than that of Sierra Madre, the valley breeze at 14 h can be expected to be larger over the Cordillera Central than over the Sierra Madre; in fact, this feature can be clearly seen in Fig. 5b. Modulated by this asymmetry of valley breeze convergence, a larger low-level wind convergence coupled with more active upward motion and precipitation is likely to be formed over western Luzon than over eastern Luzon at 14 h. In addition to these island-scale features, there are also large-scale westerly wind anomalies appearing at 14 h, covering most domains of 115–128°E from 1000 to 600 hPa (Fig. 5b). The Cordillera Central and Sierra Madre are on the windward and leeward sides of the large-scale westerly wind anomalies, respectively. The interaction between the topography and this large-scale westerly wind anomalies can lead to enhanced wind convergence over western Luzon but a suppressed wind convergence over eastern Luzon. This might also contribute to the east–west asymmetry distribution of diurnal precipitation at 14 h.

Compared to the above-mentioned features at 14 h (Fig. 5a), the low-level wind convergence displays a more east–west symmetry spatial distribution at 17 h, with a convergence center located over central Luzon. The enhancement of sea breeze convergence from 14 to 17 h may push the two upward motions over the Cordillera Central and Sierra Madre, thereby moving the motions toward each other and combining them into one big system (Fig. 5b). Consequently, positive values of the diurnal precipitation anomalies are revealed over most of Luzon at 17 h (Fig. 5a). However, compared to 17 h, valley breezes were suppressed and mountain breezes developed at 20 h. Because the mountain breezes at 20 h had only just developed and mainly appeared at low-levels, this weak feature cannot be easily seen in Fig. 5b. Thus, a more detailed illustration of the mountain breezes at 20 h has been provided in Supplementary Fig. S3. The mountain breezes over the eastern slope of the Cordillera Central mainly appeared at levels below 900 hPa (Fig. S3a); these mountain breezes meet those from the Sierra Madre, leading to a convergence with upward motion over the Cagayan Valley at 20 h (Fig. S3b-c).

In contrast, the large-scale wind anomalies over the ocean near eastern Luzon changed from westerly wind dominant during 14–17 h to easterly dominant at 20 h (Fig. 5b). These large-scale easterly wind anomalies can interact with the mountain breezes from the Sierra Madre (Fig. S3; below 950-hPa levels and around 17–18°N), leading to a weak local wind convergence and precipitation over eastern Luzon at 20 h. In contrast, the aforementioned large-scale westerly wind anomalies at 14 h remained over the ocean west of Luzon at 20 h; these anomalies interacted with the mountain breezes from the Cordillera Central, which resulted in weak precipitation over the coastal region of western Luzon at 20 h (Fig. 5 and Fig. S3). At 23 h, the large-scale easterly wind anomalies were enhanced over the ocean east of Luzon and even expanded to a status of dominance over the ocean west of Luzon (Fig. 5b). Consequently, the interactions between the topography and large-scale easterly wind anomalies can lead to a low-level wind convergence, upward motion, and precipitation formation over the ocean near eastern Luzon (the windward side) but not near western Luzon (the leeward side). At 23–02 h, the island-scale land breezes and mountain breezes over Luzon appeared to enhance (Fig. 5b), which may have further enhanced the low-level wind convergence over the ocean near eastern Luzon. Moreover, the related precipitation was enhanced and pushed further eastward (Fig. 5a).

This discussion has thus far focused on exploring the role of diurnal wind anomalies (with daily mean removed) in modulating diurnal precipitation formation over Luzon and the nearby oceans, but it has not examined whether upper-level prevailing wind (without daily mean removed) also helps in forming the eastward propagation of diurnal features. To address this issue, we added the positive values of prevailing longitudinal wind (green contours) in Fig. 5b and constructed a vertical cross-section for the distribution of prevailing longitudinal winds extending upward to 200 hPa (Supplementary Fig. S4). As noted from Fig. S4, the prevailing westerly winds (marked by green contours) mainly appeared at mid-to-upper-levels from 14–02 h. At 14–17 h, this mid-to-upper-level prevailing westerly wind couples with the low-level westerly wind over the west of the Cordillera Central. This prevailing westerly wind likely caused the precipitation system and upward motion (–Δω > 0, extending upward to 200 hPa; Fig. S4) to move eastward and cover most of Luzon at 17 h. In contrast, the prevailing westerly winds disappeared below 500 hPa from 20–02 h and thus were unlikely to play a role in facilitating the eastward propagation of diurnal precipitation at this time, when the upward motion (–Δω > 0, Fig. S4) was mainly located in the low-to-mid-levels.

To summarize the features shown in Fig. 5, we suggest that the eastward propagation of diurnal precipitation over Luzon is a result of the interaction between the topography and multiple-scale circulation changes (including island-scale LSB, mountain–valley breeze, large-scale diurnal wind anomalies, and mid-to-upper-level prevailing wind) during the day. Among these mechanisms, the impact of island-scale LSB, mountain–valley breeze, and upper-level prevailing wind are also suggested to be important factors that drive the westward propagation of diurnal precipitation over Luzon in summer^7,8,10,11^. However, unlike the above-mentioned upper-level prevailing westerly wind in spring, the upper-level prevailing wind in summer is easterly^7^. This partly explains why the propagation of diurnal precipitation is different between spring and summer.

**Figure 6:**
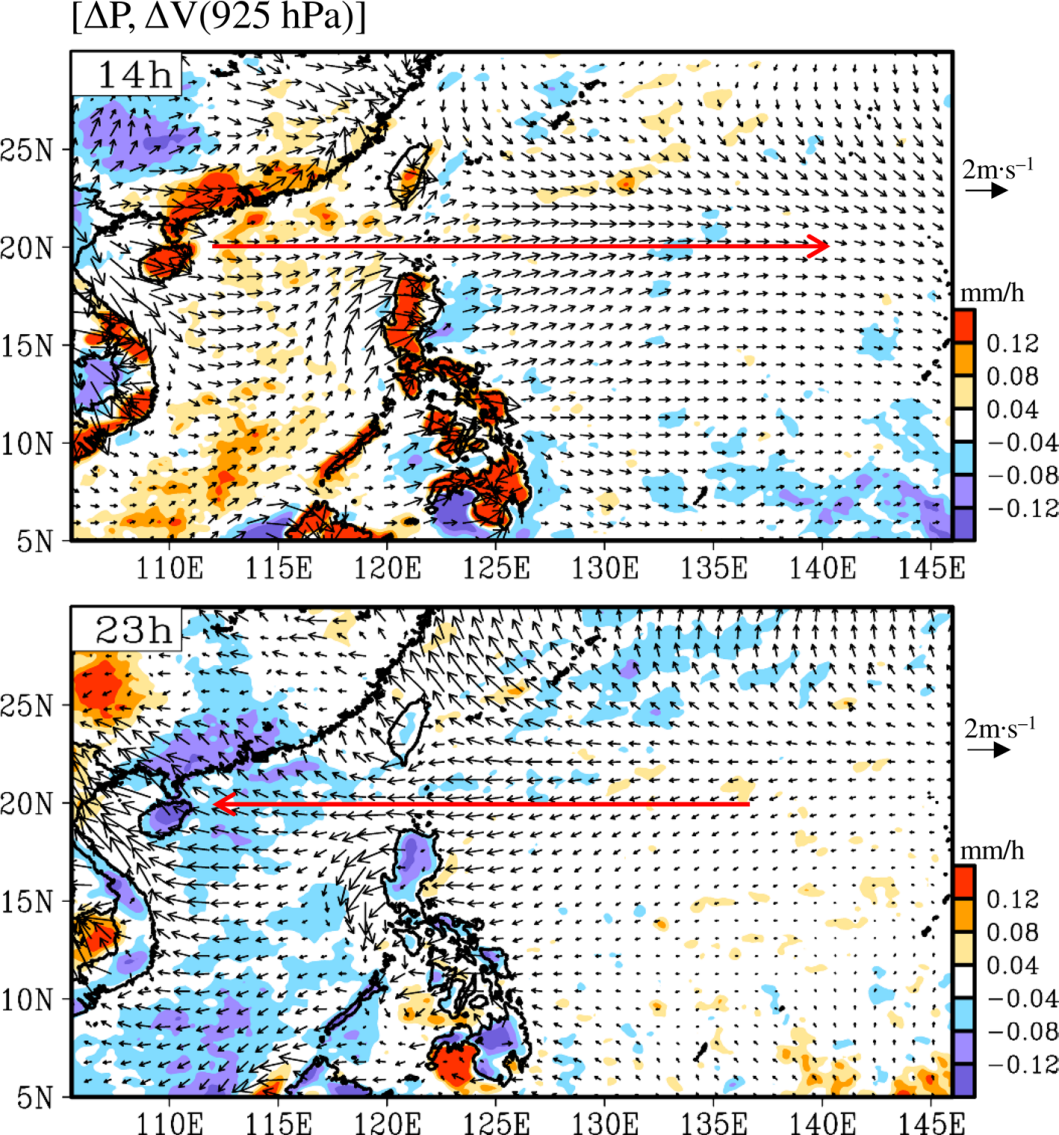
Similar to Fig. 5a, but for the enlarged domain of ΔP (shaded) and 925 hPa wind field (ΔV, vector) at 14 and 23 h (local time in the Philippines). The red arrows indicate the large-scale land–sea breeze (LSB)-like circulation feature. This figure is created using the software of GrADS v2.1.1.b0.

**Figure 7:**
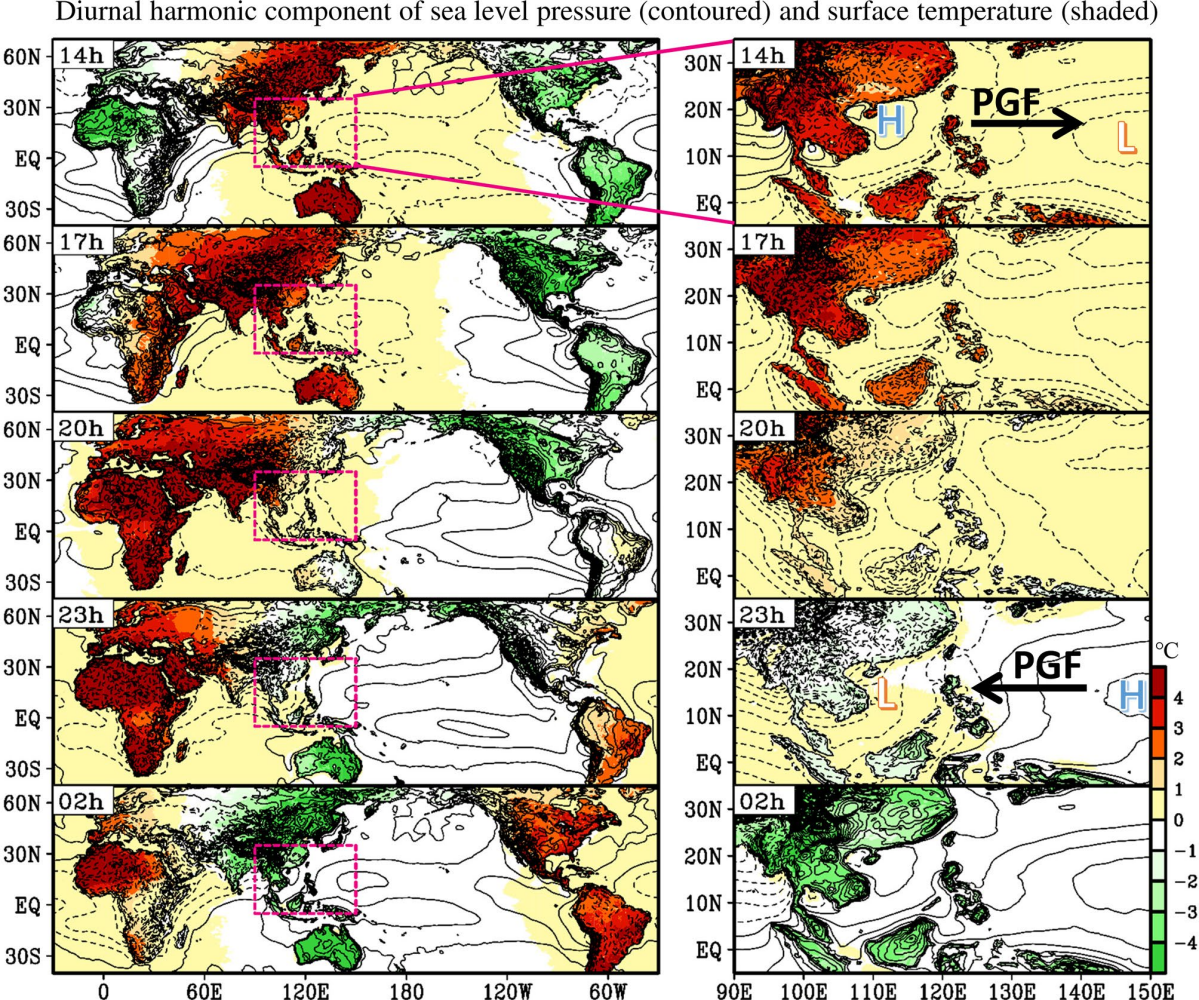
Horizontal distribution of the diurnal harmonic component of the sea level pressure (SLP, contour) and surface temperature (Ts, shaded) averaged during 2001–2019 spring. The left (right) panel represents the domain covering global longitudinal region (EAWNP region). The times (14–02 h) represent the local time in the Philippines. The contour interval is 0.1 hPa. The black arrows are added in the right panel to help indicate the direction of the pressure gradient forcing (PGF) induced by the east–west contrast of high pressure system (denoted by H) and low pressure system (denoted by L). This figure is created using the software of GrADS v2.1.1.b0.

Notably, the role of large-scale diurnal wind anomalies, which are important in explaining the eastward propagation of precipitation in spring during 20–02 h, has not been discussed in previous studies that focused on the formation of diurnal precipitation over Luzon. Therefore, the causes of the large-scale diurnal wind anomalies (shown in Fig. 5) must be determined. To clarify this phenomenon, Fig. 6 shows an enlarged domain of Fig. 5a for two selected time steps: 14 and 23 h. The diurnal wind anomalies changed from westerly covering 105–140°E (approximately 3000 km) at 14 h to easterly covering 105–135°E at 23 h. This feature appears to be the large-scale LSB-like circulation over the EAWNP region, as noted by Huang et al.^36^, who suggested that the diurnal variation of the global pressure tidal wave is the main reason for this large-scale LSB-like circulation. Huang et al.^36^ demonstrated that the anomalies of diurnal wind fields over the EAWNP region are mainly dominated by their diurnal harmonic component and not their non-diurnal harmonic component. Similar to their study, we have noted that the large-scale LSB-like circulation at 14 h (Supplementary Fig. S5a) is mostly explained by its diurnal harmonic component (Fig. S5b) and less by the non-diurnal harmonic component (Fig. S5c). Moreover, we have also noted that the eastward propagation of the diurnal convergence over Luzon (Fig. S6a) is mainly explained by its diurnal harmonic component (Fig. S6b) and less by the non-diurnal harmonic component (Fig. S6c). Therefore, following the work of Huang et al.^36^, we have performed an examination using the diurnal harmonic component (Fig. 7) to further clarify the possible relationship between the large-scale diurnal wind anomalies shown in Fig. 5b and the global pressure tidal wave.

Figure 7 shows the spatiotemporal evolution of the diurnal harmonic component of the sea level pressure and surface temperature over the global domain (left panel). Overall, the surface heated by the sun moves westward over time, and the sea level pressure decreases (increases) after the surface temperature increases (decreases). To focus on the EAWNP region, we enlarged the regional feature (right panel). At 14 h, the surface temperature of the Asian land was higher than the ocean, while the positive value of sea level pressure moved to the South China Sea (110°E) and the Bay of Bengal (90°E); however, the negative value of sea level pressure was over the Central Pacific (150°E). The distribution of sea level pressure causes the pressure gradient force from the South China Sea to the Central Pacific, resulting in large-scale westerly diurnal wind anomalies covering 110–150°E. In contrast, at 23 h, the surface temperature of the Asian land was colder than that of the ocean, while the sea level pressure on the Asian land and the South China Sea (110°E) was negative; however, sea level pressure over the Central Pacific (150°E) was positive. Therefore, the pressure gradient force is from the Central Pacific to the South China Sea, resulting in large-scale easterly diurnal wind anomalies covering 110–150°E. These findings indicate that the large-scale westerly (easterly) diurnal wind anomalies over the ocean at 14 h (23 h) shown in Fig. 5 is a part of the large-scale LSB-like circulation caused by the diurnal change of large-scale pressure tidal waves.

## Conclusions

**Figure 8:**
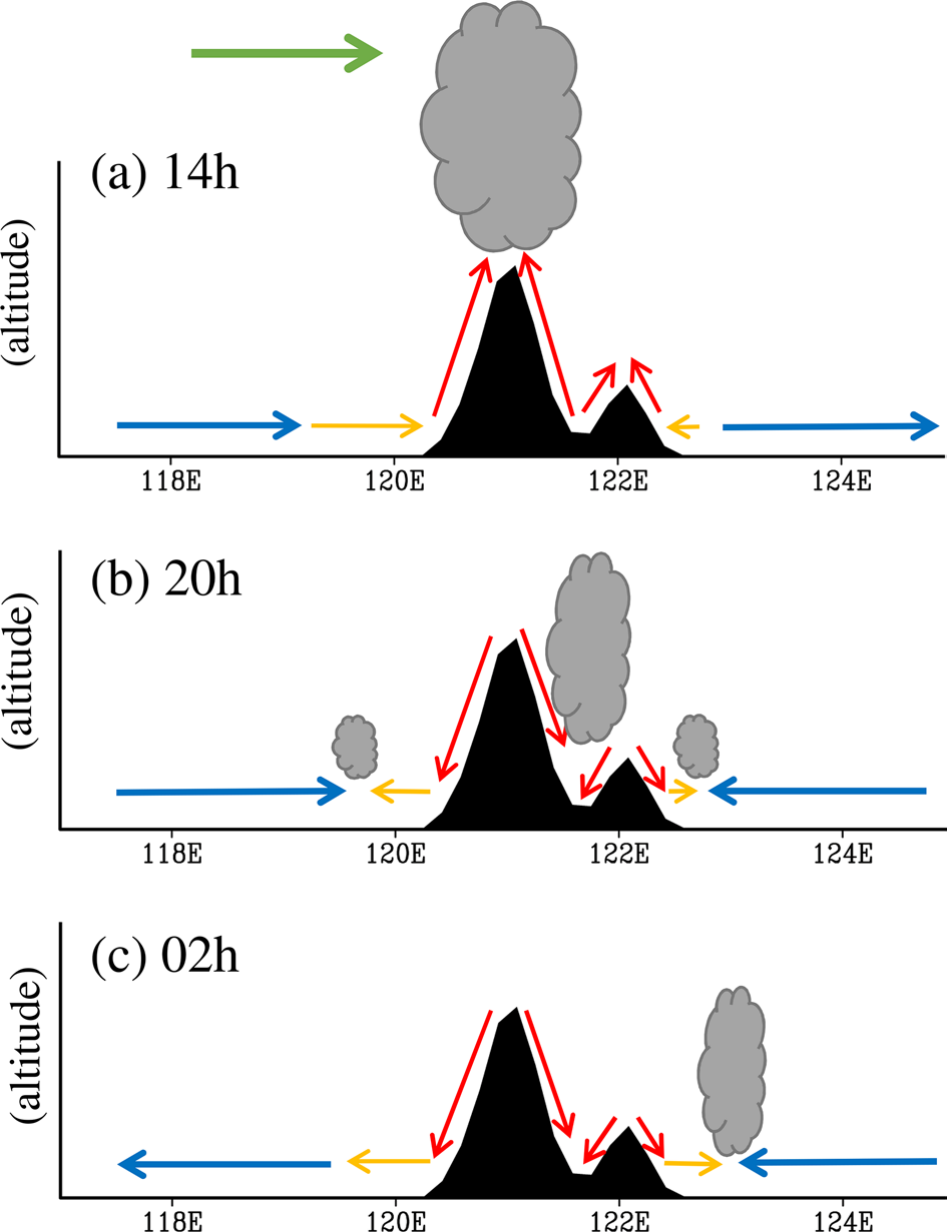
Interaction between topography (black area) and multiple timescale circulations (arrows) over Luzon and the nearby oceans at local time (**a**) 14 h, (**b**) 20 h, and (**c**) 02 h. The red, yellow, blue, and green arrows represent the directions of the mountain–valley breeze, the island-scale land–sea breezes (LSB), the large-scale LSB-like circulation, and the mid-to-upper-level prevailing westerly wind, respectively. The formations of cloud (gray) induced by the convergence of wind fields are added to indicate the eastward propagation of diurnal precipitation shown in Fig. 5.

This study examined the spatiotemporal characteristics of spring diurnal precipitation over Luzon and the nearby oceans, along with the related maintenance mechanisms. We analyzed the satellite precipitation from TRMM and IMERG during 2001–2019 for MAM. The results show that the climatological spring diurnal precipitation over Luzon is mainly dominated by an eastward propagating feature. This propagation direction is opposite to the prevailing low-level easterly wind in spring and differs from the well-known westward propagation direction of diurnal precipitation over Luzon in summer. Examining the related formation mechanisms, our study findings show that the causes of eastward propagation diurnal precipitation can be attributed to the interaction between topography and multiple-scale atmospheric circulation changes (including island-scale LSB, mountain–valley breeze, large-scale LSB-like circulation, and mid-to-upper-level prevailing westerly wind). A schematic diagram is shown in Fig. 8 to summarize the related mechanisms explained below.

At 14 h (Fig. 8a), the circulation over Luzon was dominated by large-scale low-level westerly diurnal wind anomalies (blue arrow), island-scale sea breeze (yellow arrow), valley breeze (red arrow), and mid-to-upper-level prevailing westerly wind (green arrow). All these dynamic factors interacted with the topography, leading to large precipitation over western Luzon than eastern Luzon at 14 h, which then expanded from western Luzon to cover most of Luzon during 14–17 h. At 20 h (Fig. 8b), the circulation changed to become dominated by large-scale low-level easterly diurnal wind anomalies over east of Luzon (blue arrow), island-scale land breeze (yellow arrow), and mountain breeze (red arrow). The interaction between these factors led to low-level wind convergence and precipitation mainly over the eastern Luzon and the nearby oceans. At 02 h (Fig. 8c), the circulation was also dominated by large-scale low-level easterly diurnal wind anomalies (blue arrow), island-scale land breeze (yellow arrow), and mountain breeze (red arrow). However, compared to the case at 20 h, the island-scale land breeze and mountain breeze over Luzon were enhanced at 02 h; this could further enhance the low-level wind convergence over the ocean near eastern Luzon, and the related precipitation was also enhanced and pushed eastward. All these features together lead to the eastward propagation of diurnal precipitation features seen over Luzon and the nearby oceans in spring.

Notably, this study is the first to demonstrate the role of large-scale LSB-like circulation in modulating diurnal precipitation formation over Luzon. Our findings provide new insights into the modulation of the local weather in Luzon by large-scale circulation changes. However, there might be other possible formation mechanisms (e.g., gravity wave^16,25^, thermal instability, cold pool outflow^11,26^, etc.) contributing to the eastward propagation feature that have not been included in our discussion; this must be investigated further.

## Supplementary Information

Supplementary Figures
