# Supplementary material for: Impact of multiple-scale circulation interactions on the spring diurnal precipitation over Luzon
Source: Sci Rep. 2021 May 11;11:9937. doi: 10.1038/s41598-021-89392-0 (PMC8113498; doi:10.1038/s41598-021-89392-0)
Supplement: Supplementary file 1 — Supplementary Figures [file 41598_2021_89392_MOESM1_ESM.pdf]

Supplementary Information for

Impact of multiple-scale circulation interactions on the spring diurnal precipitation over Luzon

Authors:
Cheng-An Lee[1], Wan-Ru Huang[1]*, Ya-Hui Chang[1], and Shih-Ming Huang[2]

Affiliation:
[1]Department of Earth Sciences, National Taiwan Normal University, Taipei, Taiwan R.O.C.
[2]Department of Atmospheric Sciences, Chinese Culture University, Taipei, Taiwan R.O.C.

*Corresponding author E-mail: wrhuang@ntnu.edu.tw

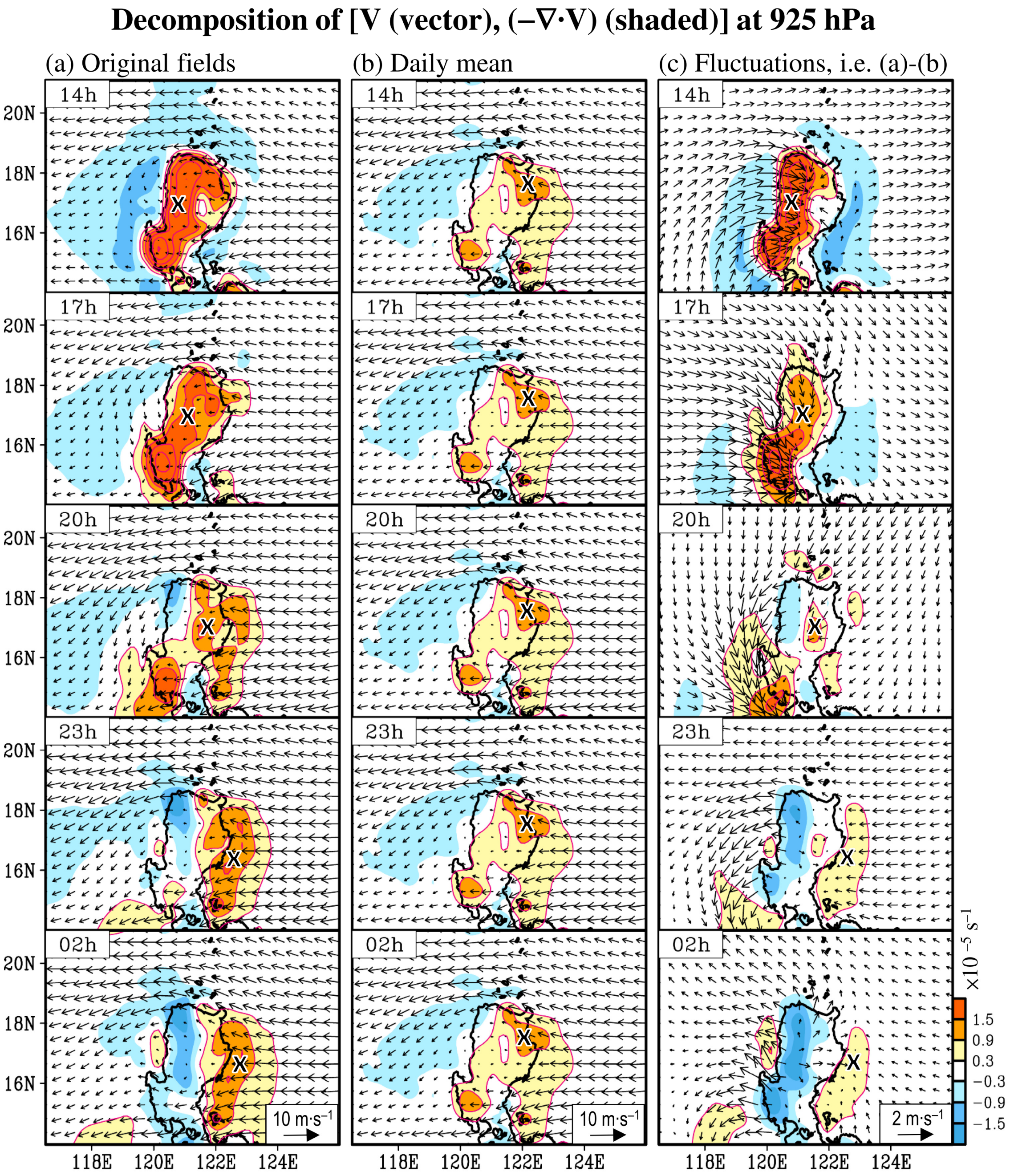

Fig. S1 (a) Horizontal distribution of low-level wind fields at 925 hPa (vector) superimposed with its related wind convergence (shaded, positive are marked by contour) extracted from ERA5 and averaged for spring from 2001–2019. (b) and (c) are related to (a), but represent the daily mean and fluctuations (i.e., anomalies with the daily mean removed), respectively. The times (14–02 h) represent the local time in the Philippines. In (a) and (c), the symbol "x" indicates the eastward propagation of wind convergence. In (b), the symbol "x" indicates the location of maximum wind convergence between 16–18°N. This figure is created using the software of Grid Analysis and Display System version 2.1.1.b0 (hereafter GrADS v2.1.1.b0), available at http://cola.gmu.edu/grads/downloads.php.

Decomposition of low-level wind fields at 14 h

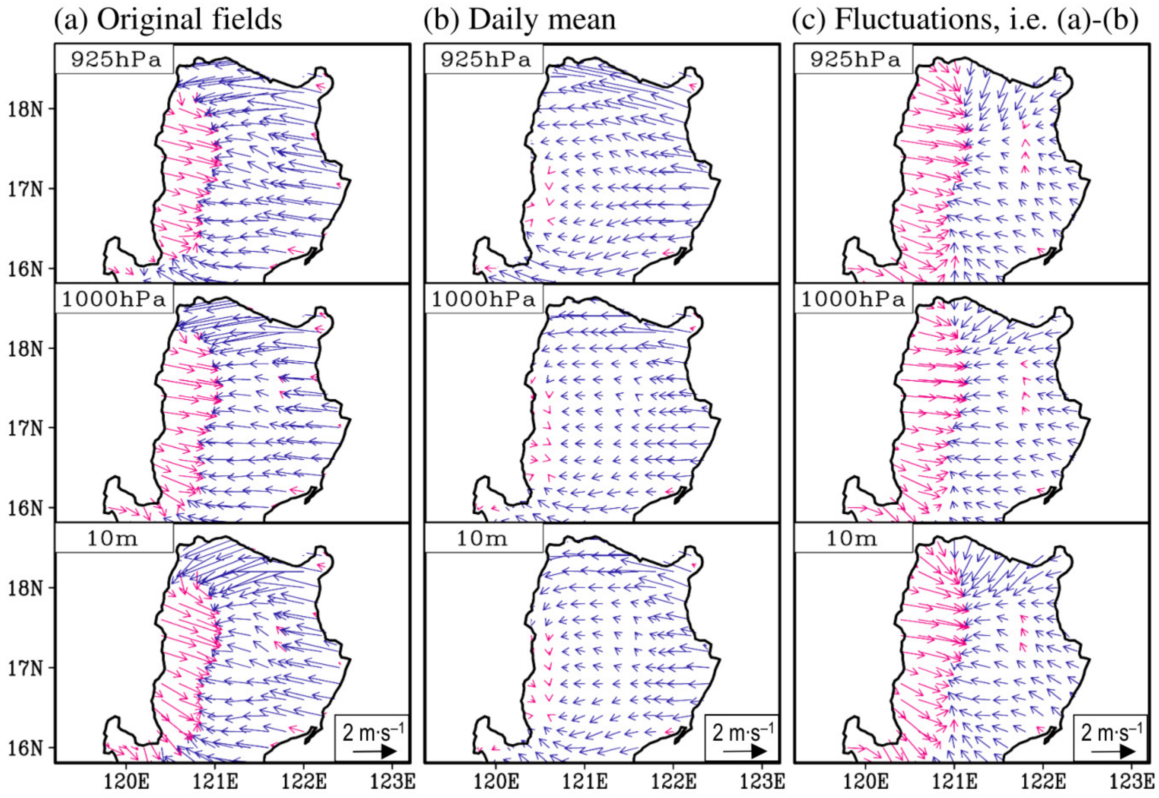

Fig. S2 Similar to Fig. S1, but only for the decomposition of low-level wind fields (925 hPa, 1000 hPa, and 10 m) over the land areas at 14 h (local time in the Philippines): (a) original component, (b) daily mean, and (c) fluctuations. In (a)-(c), the wind vectors are plotted using the same scale. The positive and negative value of longitudinal winds are shown in red and blue, respectively. This figure is created using the software of GrADS v2.1.1.b0.

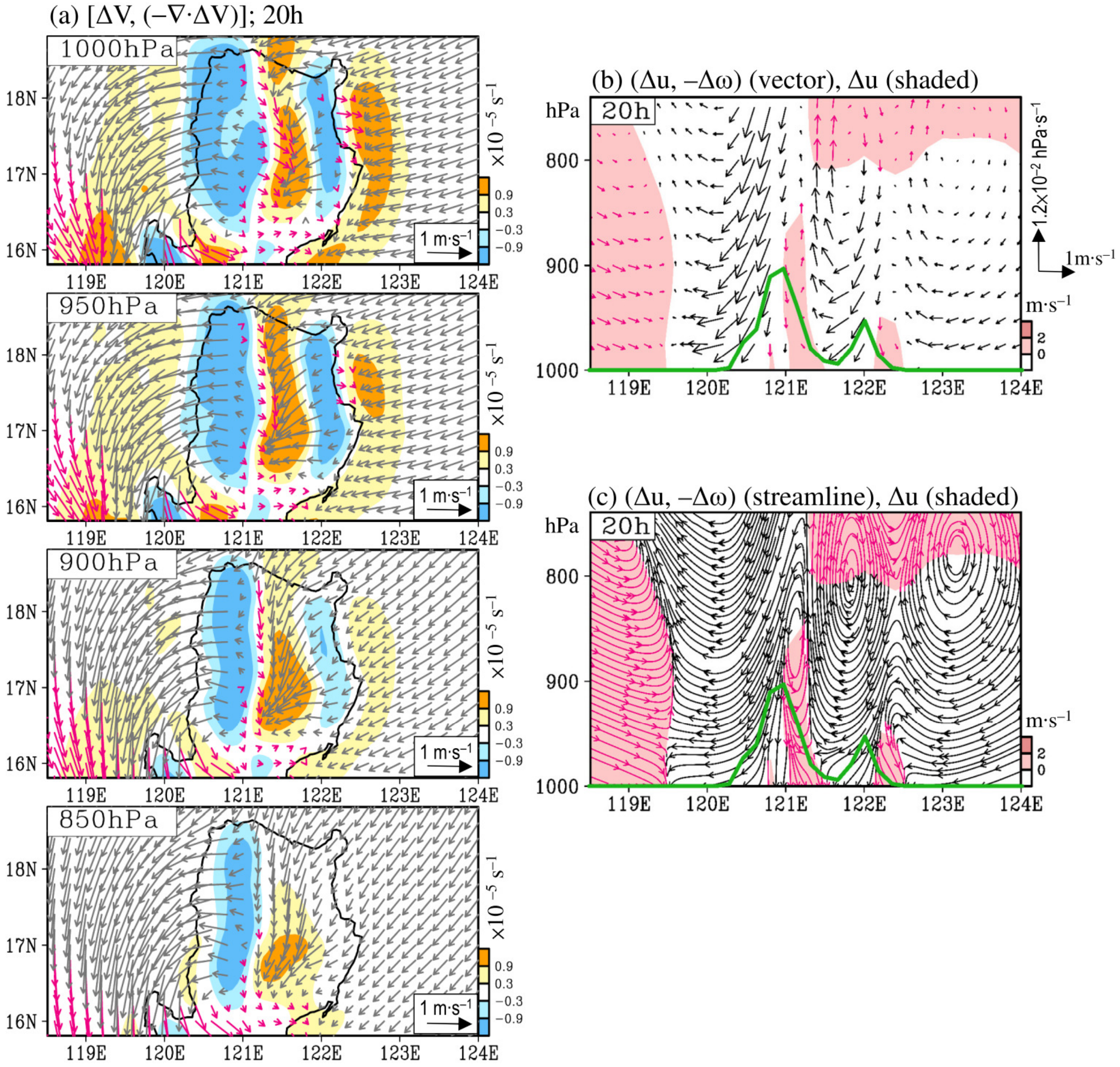

Fig. S3 (a) Horizontal distribution of diurnal anomalies of wind vectors at four low-levels (1000, 950, 900, and 850 hPa); this distribution is superimposed with related wind convergences (shaded) for the specific time step at 20 h (local time in the Philippines) averaged from 2001–2019 MAM. The vector in red represents the positive value of longitudinal wind (i.e., westerly). (b) represents the vertical cross-section of diurnal anomalies for circulation at 20 h, averaged between 17–18°N, zoomed in from Fig. 5b. To better show the mountain–valley breeze, (c) is related to (b) but is plotted via streamline. In (b)–(c) mountains are shown with the green contour, and the positive value of the longitudinal wind is shown in red. This figure is created using the software of GrADS v2.1.1.b0.

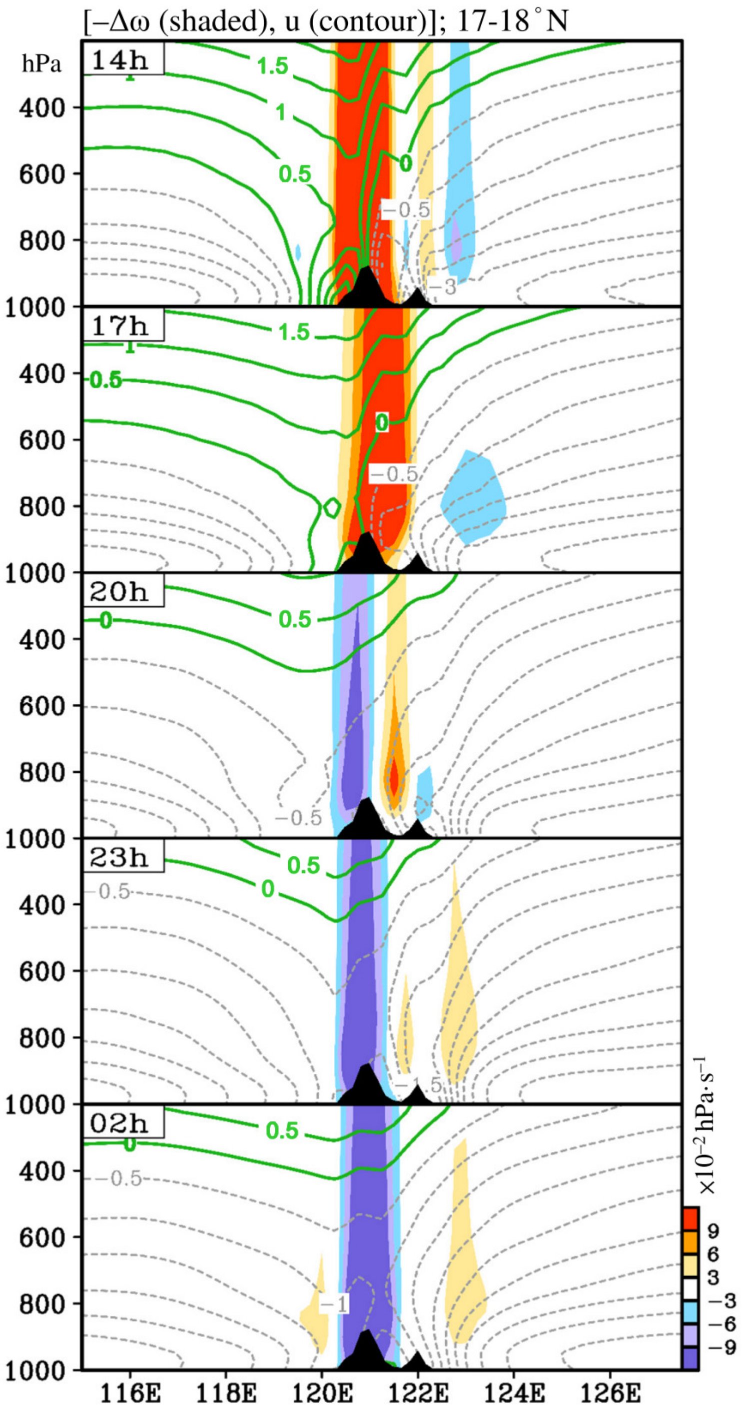

Fig. S4 A vertical cross-section of the original longitudinal wind (u, contour; positive are marked by green color), superimposed with the anomalies of diurnal vertical motion (shaded), averaged between 17–18°N and from 2001–2019 MAM. The contour interval of u is 0.5 m/s. The times (14–02 h) represent the local time in the Philippines. This figure is created using the software of GrADS v2.1.1.b0.

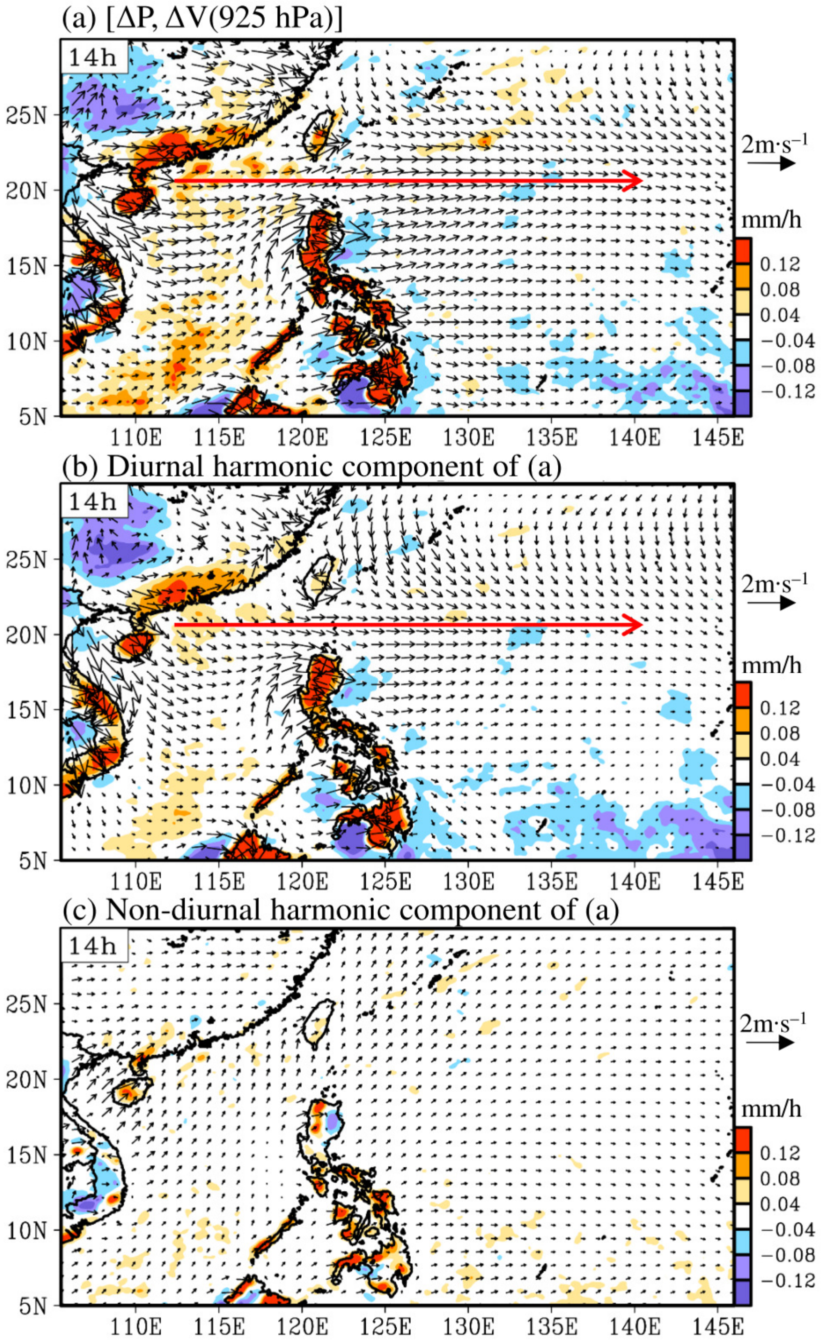

Fig. S5 (a) Horizontal distribution of diurnal anomalies (daily mean removed; denoted as Δ) of precipitation (ΔP, shaded) with the 925 hPa wind field (ΔV, vector) at 14 h (local time in the Philippines), averaged for 2001–2019 MAM. (b) is the diurnal harmonic component of (a). (c) is the non-diurnal harmonic component of (a), which is obtained by (a) minus (b). This figure is created using the software of GrADS v2.1.1.b0.

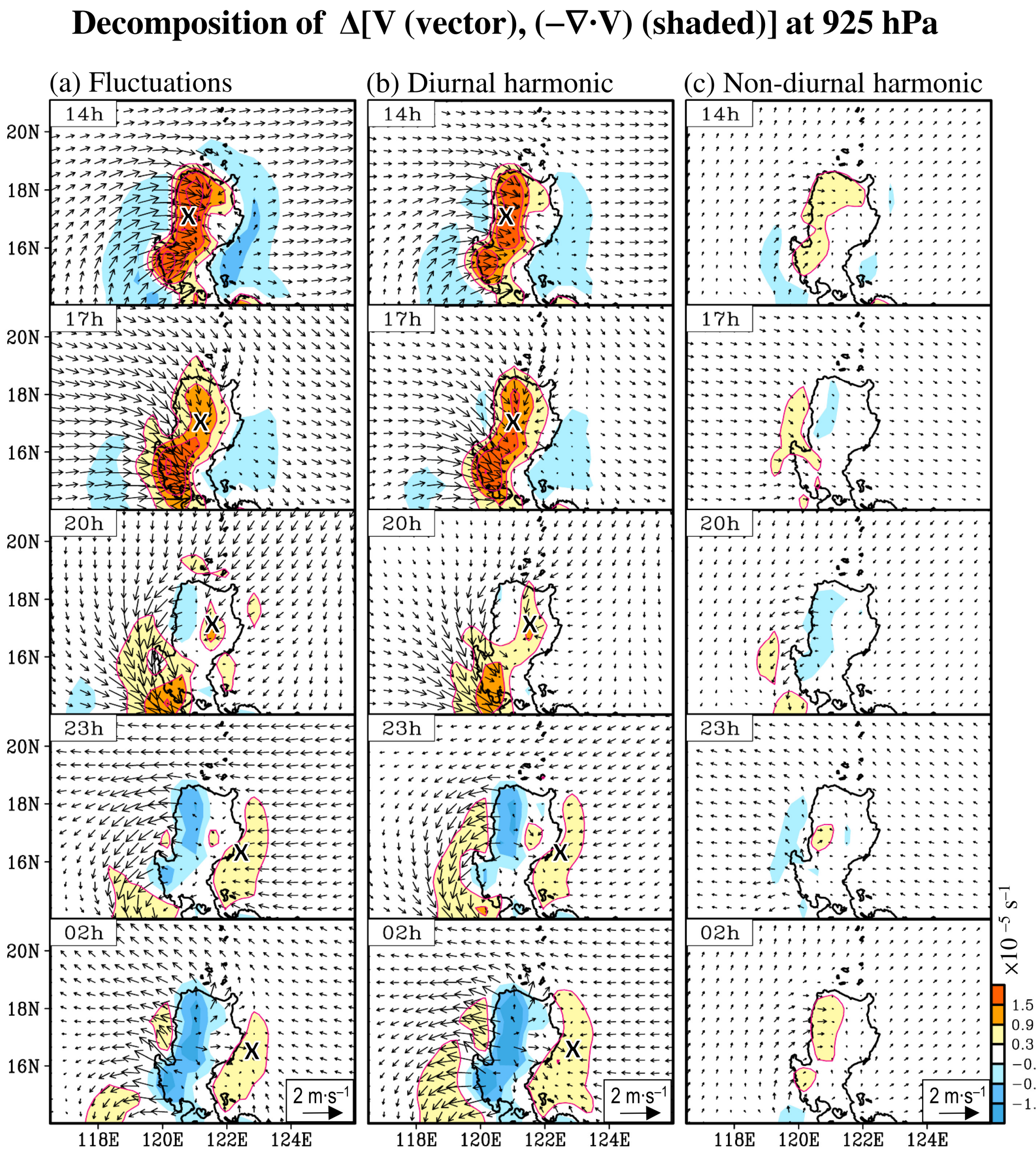

Fig. S6 (a) is similar to Fig. S1c but represents the horizontal distribution of diurnal anomalies (daily mean removed; denoted as Δ) of the 925 hPa wind field (ΔV, vector) with the related wind convergence (shaded, positive are marked by contour), averaged for 2001–2019 MAM. (b) is the diurnal harmonic component of (a). (c) is the non-diurnal harmonic component of (a), which is obtained by (a) minus (b). The symbol "x" indicates the eastward propagation of wind convergence. This figure is created using the software of GrADS v2.1.1.b0.
